# Statistical analysis of twenty years (1993 to 2012) of data from mainland China’s first intervention center for children with autism spectrum disorder

**DOI:** 10.1186/2040-2392-5-52

**Published:** 2014-11-12

**Authors:** Wei-Zhen Zhou, Adam Yongxin Ye, Zhong-Kai Sun, Hope Huiping Tian, Tad Zhengzhang Pu, Yu-Yu Wu, Dan-Dan Wang, Ming-Zhen Zhao, Shu-Juan Lu, Chang-Hong Yang, Liping Wei

**Affiliations:** Center for Bioinformatics, State Key Laboratory of Protein and Plant Gene Research, School of Life Sciences, Peking University, No.5 Yiheyuan Road, Haidian District, Beijing, 100871 China; National Institute of Biological Sciences, No.7 Science Park Road, Zhongguancun Life Science Park, Beijing, 102206 China; Beijing Stars and Rain Education Institute for Autism, No.18 Shuangqiao East Road, Beijing, 100121 China; Shanghai Parkway Health, No.51 Hongfeng Road, Jin Qiao, Pudong, Shanghai, 201206 China; Yuning Psychiatry Clinic, No.6, Section 2, Fuxing South Road, Da’an District, Taipei, 10664 Taiwan

**Keywords:** Autism spectrum disorder, Diagnosis, Intervention, Parental age, China

## Abstract

**Background:**

Autism spectrum disorder (ASD) is characterized by persistent deficits in social communication and interaction, and restrictive and repetitive patterns of behavior, interests or activities. This study aimed to analyze trends in ASD diagnosis and intervention in 20 years of data from the Beijing Stars and Rain Education Institute for Autism (SR), the first autism intervention center in mainland China, and from a recent survey of members of the Heart Alliance, an industry association of autism intervention centers in China.

**Methods:**

We analyzed the registration data at the SR from 1993 to 2012 for a total of 2,222 children who had a parent-reported diagnosis of ASD and 612 of ‘autistic tendencies’. Most of the children who were the primary focus of our analyses were age six and under. We also analyzed results of a survey we conducted in 2013 of 100 member centers of the Heart Alliance. Generalized Estimating Equations, multiple linear regression and the Mann-Whitney test were used for data analysis. Statistically significant findings are reported here.

**Results:**

The number of hospitals where SR children received their diagnosis increased from several in the early 1990s to 276 at present. The proportion of ‘autistic tendencies’ diagnosis increased 2.04-fold from 1998 to 2012 and was higher for children diagnosed at a younger age. The mean age at first diagnosis of ASD or ‘autistic tendencies’ decreased by 0.27 years every decade. A higher level of parental education was statistically significantly associated with an earlier diagnosis of the child. The mean parental age at childbirth increased by about 1.48 years per decade, and the mean maternal age was 1.40 and 2.10 years higher than that in the national population censuses of 2000 and 2010, respectively. At the time of the survey 3,957 children with ASD were being trained at the 100 autism intervention centers. Ninety-seven of these centers opened after the year 2000. Economically underdeveloped regions are still underserved.

**Conclusions:**

This study revealed encouraging trends and remaining challenges in ASD diagnosis and intervention among children at the SR over the past 20 years and the 100 autism intervention centers in China at present.

**Electronic supplementary material:**

The online version of this article (doi:10.1186/2040-2392-5-52) contains supplementary material, which is available to authorized users.

## Background

Autism spectrum disorder (ASD) is a neurodevelopmental disorder characterized by persistent deficits in social communication and social interaction, and restrictive and repetitive patterns of behavior, interests or activities [1]. This disorder was not recognized in China until 1982 when the first cases were reported by Kuotai Tao [2]. Autism-associated disability has become the most prevalent mental disability among Chinese children [3]. No nationwide prevalence survey of ASD in China has been published yet. Based on recent prevalence estimates of ASD of 1 in 110 to 1 in 50 in the US [4–7] and 1 in 38 in South Korea [8], there may be as many as 10 to 20 million people in China affected by ASD.

The Chinese health system for children with ASD has previously been reviewed [9–13]. Clinical diagnoses are made according to the Chinese Classification and Diagnosis Criteria of Mental Disorders, 3^rd^ edition (CCMD-3) [14], which is based on the Diagnostic and Statistical Manual of Mental Disorders, Fourth Edition (DSM-IV) [15] and the International Statistical Classification of Diseases and Related Health Problems, 10th Revision (ICD-10) [16]. For children under three and those with high-functioning autism, making definitive diagnosis early could be challenging. Doctors in China sometimes give out the diagnosis of ‘autistic tendencies’ or give no diagnosis, but encourage the parents to seek intervention for the children as soon as possible without waiting for a definitive clinical diagnosis to be made [9].

Intervention is provided primarily by private centers and a smaller number of public centers in China [11, 12]. The Beijing Stars and Rain Education Institute for Autism (SR), founded in 1993, was the first autism center in China. From 1993 to 1995, the SR provided one-on-one intervention for children with ASD. In 1996 when the demand for service quickly became too high and few other intervention services were available in China at the time, the SR changed its service mode to provide an 11-week-long training service of Applied Behavior Analysis (ABA) for parents and children together, and since 1998, the age of the children at the time of registration has been restricted to six and under [9, 17, 18]. Space was offered on a first-come-first-serve basis. Children with other neuropsychiatric disorders or symptoms who may also benefit from ABA training were discouraged but not denied admission. From 1993 to 2012, 5,143 children were registered at the SR. Until now, data on this valuable ASD population had not been analyzed.

Behavior intervention as soon as possible can have positive impact on the outcome of children with ASD [19–21]. Early intervention is conditional upon early diagnosis [22, 23]. Routine neuropsychological screenings of children are not yet available in China. A child is usually taken to visit a doctor when parents or teachers notice unusual behavior in him/her. Data from the SR enabled us to study the trends in the age of diagnosis over the two decades and parental factors that may contribute to early diagnosis of the SR children.

Advanced maternal and paternal ages have been associated with the risk of ASD in some, but not all, studies [24–31]. Findings on the association between maternal age and the risk of autism are more varied than findings on the association between paternal age and the risk of autism [32, 33]. In the Chinese population, only one case-control study of 190 Chinese children of Han ethnicity found that advanced paternal age (>30 years old) but not maternal age was statistically significantly associated with the risk of autism [34]. Data from the SR enabled us to analyze the parental age at childbirth of the children from the SR and compare it to the national census of the general population.

It has been estimated that at present there are around 1,000 ASD intervention centers in China (H Wen, personal communication). In 2005, the SR founded Heart Alliance, an industry association of autism intervention centers [35]. Heart Alliance had 150 member centers at the time of this study (2013) and has 230 member centers today. Previous research on autism intervention in China covered at most a few centers, giving a description based on interviews with the centers’ directors and teachers and direct observations [9, 11–13]. Larger surveys had not yet been conducted. Here, we conducted a survey of 100 member centers of the Heart Alliance and analyzed the survey results. Our study covered about 10% of all autism intervention centers in China, and is the largest such study so far.

## Methods

This study was approved by the Peking University Institutional Review Board.

The SR data were de-identified before being made available for analyses: all personal-identifying information such as name, parents’ names, and contact information, including phone numbers, email addresses and street address were removed; each record was assigned an irreversible unique identifier. Informed consent was not required for this de-identified population.

### The Beijing Stars and Rain Education Institute for Autism historical data

From 1993 to 2012, a total of 5,143 children were registered in the SR registry. The SR staff collected registration questionnaires from the child’s caregiver. All questionnaires were assigned a file number and entered into an electronic registration system. Two versions of the questionnaires have been used. The second version was used since 2011 and a total of 693 children used the new version. Although there were small differences between the two versions, both versions consisted of the following five sections.

The first section queried demographic information about the child such as gender, birth date, and current age, and information about both parents such as age when married, education level and occupation. The second section included questions about prenatal conditions such as the length of pregnancy, and complications, illnesses and medications taken during pregnancy. The third section consisted of questions about perinatal conditions and developmental milestones of the child. The fourth section included questions about the medical history of the child where the caregiver was asked to fill in the blanks with the child’s diagnosis, age of diagnosis and hospital of diagnosis. It also included questions about the medical history of the extended family. The last section included ASD assessments such as the Clancy Autism Behavior Scale [36].

De-identified data were retrieved and saved in Excel files and preprocessed as described below. Parent-reported clinical diagnoses were classified as one of three categories: ‘ASD’, ‘autistic tendencies’ and ‘other’. The ‘ASD’ category included diagnoses of Autism, Asperger Syndrome, Pervasive Developmental Disorder, Not Otherwise Specified (PDD-NOS) or Rett Syndrome. ‘Autistic tendencies’ included diagnoses of ‘suspected autism’, ‘autistic-like case’, or ‘having autistic tendencies’. Diagnoses of other diseases and those missing information on diagnosis were classified into the ‘other’ category. A small number of children had received more than one diagnosis over time; children with at least one diagnosis of ASD were assigned to ‘ASD’ group; those with at least one diagnosis of ‘autistic tendencies’ but no diagnosis of ASD were assigned to the ‘autistic tendencies’ group. In total, 2,222 children had a parent-reported clinical diagnosis of ASD, 612 of ‘autistic tendencies’, and 2,309 was assigned to the ‘other’ group including 203 with a parent-reported clinical diagnosis of other neuropsychiatric disorders and 2,106 missing parent-reported clinical diagnosis.

Age at diagnosis was calculated by subtracting the date of birth from the date of diagnosis. Maternal and paternal ages at childbirth were calculated from the filing date, child’s birth date and parents’ current age. Parental education was classified into five levels: middle school or lower, high school, junior college, regular college, and graduate, and this ordinal variable was coded as an interval variable from one to five with equal intervals in the regression models. The ‘middle school or lower’ category included no schooling, literacy classes, primary school and middle school. Special secondary schools and high schools were both classified as the ‘high school’ category.

### Survey of autism intervention centers

We contacted via email the directors of 101 autism intervention centers, including the SR, and invited them to complete an 18-item survey. These centers were all members of the Heart Alliance and had previously agreed to participate in our research. In the absence of response to the initial contact, we contacted the directors by phone two days later. One hundred directors completed the survey. We checked all of the returned answers for quality control and administered a telephone interview to confirm missing or incorrect values. The entire survey was conducted from 29 January to 10 March 2013. The survey queried information about each center’s date of establishment, location, business type, number of children, age range of the children, diagnoses of the children, number of teachers, training methods and service mode.

### Confirmation of parent-reported clinical diagnosis in a group of 42 children

A group of 42 children from the SR with a parent-reported clinical diagnosis of ASD or ‘autistic tendencies’ participated in our Autism Genetic Research Project, by the end of 2012. In the Autism Genetic Research Project, we have the Peking University Institutional Review Board approval to access the full records of the children as well as assessment of the children with the Autism Diagnostic Interview, Revised (ADI-R) [37], by trained raters with reliability and clinical re-diagnosis by child psychiatrists of the children via direct observations and interview according to the Diagnostic and Statistical Manual of Mental Disorders, Fourth edition, text revised (DSM-IV-TR) [38]. Patient content was obtained from parents of all children. We compared the parent-reported clinical diagnosis with results of the ADI-R assessments and clinical reassessment by child psychiatrists. The ADI-R raters and the child psychiatrists were not blinded to parent-reported diagnoses.

The ADI-R algorithm comprises 42 items based on the DSM-IV-TR evaluating the social behavior, communication, repetitive behavior and age of onset [37]. Since there is no standard cutoff of the ADI-R for ASD, we adopted the criteria used by both the Simons Simplex Collection [39] and the Collaborative Programs for Excellence in Autism [40]. A child was classified as having ASD if she/he met the standard cutoff on the social and communication domains, or scored within two points of the cutoff on either the social or communication domains, or scored within one point on both domains.

### Data analysis

We analyzed the number of registered children each year, their geographical origin, and sex ratio using data on all 5,143 children who were registered at the SR from 1993 to 2012. All other subsequent statistical analyses were based only on the children with parent-reported clinical diagnoses of ASD or ‘autistic tendencies’. As mentioned in the Background section, the SR changed the admission criteria in 1998 to admit only children age six and under. Thus, in all analyses related to diagnosis, we removed data on children more than six years of age for consistency. For analyses with binned variables, such as the diagnosis year, the birth year, and the binned age at diagnosis, we removed bins with samples size less than 30 to ensure the robustness of our results.

We identified the hospitals where the SR children received their ASD or ‘autistic tendencies’ diagnosis. The number of hospitals and the proportion of diagnoses made by each hospital were investigated and grouped by hospital type and the administrative level of the cities in which they were located.

We calculated the proportion of ‘autistic tendencies’ diagnoses among the diagnoses of ASD and ‘autistic tendencies’, and analyzed whether this proportion increased from 1998 to 2012 and whether children diagnosed at a younger age were more likely to receive an ‘autistic tendencies’ diagnosis. We used the Generalized Estimating Equations (GEE [41]; geeglm function in the geepack package [42]) to fit a model with diagnosis year and diagnosis age as predictor variables, and the diagnosis of ‘autistic tendencies’ or not as a binary response variable. Because a child may have received more than one diagnosis over the years and these diagnoses were not independent of each other, the child’s unique ID was introduced in the GEE model as a cluster effect variable [see Additional file 1: Table S1].

We analyzed the trends in the age at first diagnosis over time. We built three linear regression models with the age at first diagnosis as the response variable and diagnosis year as the predictor variable for the diagnoses of ASD, ‘autistic tendencies’, and ASD-or-autistic tendencies, respectively. To determine whether the trends could be explained by increased diagnosis of ‘autistic tendencies’ over time and to control for it, we built a multiple linear regression model with the age at first diagnosis as the response variable and two predictor variables--the diagnosis year and the binary variable ‘autistic tendencies’ [see Additional file 1: Table S2].

We tested whether an association existed between the maternal or paternal education level and the child’s age at first diagnosis. For this purpose, two multiple linear regression models were built, both with the age at first diagnosis as the dependent variable; one model used the year of diagnosis, maternal age at childbirth, maternal education level as predictor variables, and the other used the year of diagnosis, paternal age at childbirth, paternal education level as predictor variables [see Additional file 1: Table S3A and S3B]. We added the year of diagnosis in the model as a potential confounder because it is associated with both age at first diagnosis and parental education level. We calculated the Spearman’s correlation coefficients and *P* values between the variables [see Additional file 1: Table S3C]. Children diagnosed with ASD or ‘autistic tendencies’ and who were age six and under at time of registration were included in the analyses, excluding children with first diagnosis year before 1999 due to the small sample size each year (less than 30).

We divided the children with a parent-reported clinical diagnosis of ASD and ‘autistic tendencies’ into 26 birth cohorts based on their year of birth. Eight cohorts were excluded from the analyses due to the small sample sizes (less than 30). The trends in paternal and maternal age at childbirth from 1993 to 2010 were analyzed with two multiple linear regression models. One used the maternal age at childbirth as the response variable, and the birth year and maternal education level as predictor variables. The other model used the paternal age at childbirth as the response variable, and the birth year and paternal education level as predictor variables [see Additional file 1: Table S4].

We compared the maternal age at childbirth in the SR population against the general population. Maternal ages at childbirth in the general population between 1 November 1999 and 31 October 2000 and between 1 November 2009 and 31 October 2010 were extracted from Chinese census data for the years 2000 and 2010, respectively, provided by the State Statistical Bureau of China [43, 44]. A comparison was done by Mann-Whitney test. To avoid confounding effects from maternal education, linear regression models were fit to each education level of the mother. Because the number of the SR children in each subgroup was small, we used the ages with confidence intervals predicted by the fitted linear regression for comparison.

All statistical analyses were conducted using R 3.1.0. The model-wise complete case approach was used to handle missing data, whereby those with missing data for a given model were excluded in that analysis. The 95% confidence interval (CI) was calculated by the point estimate ±1.96* standard error, and *P* values <0.05 were considered statistically significant. To examine the goodness of fit of the multiple linear regression models, we performed residual analysis and found no concerning patterns [see Additional file 2: Figure S1]; we also examined the variance inflation factors (VIFs) and found no large VIFs [see Additional file 1: Table S2, S3, S4], demonstrating that the models were appropriate.

## Results

### Characteristics of children registered at the Beijing Stars and Rain Education Institute for Autism, 1993 to 2012

**Figure 1:**
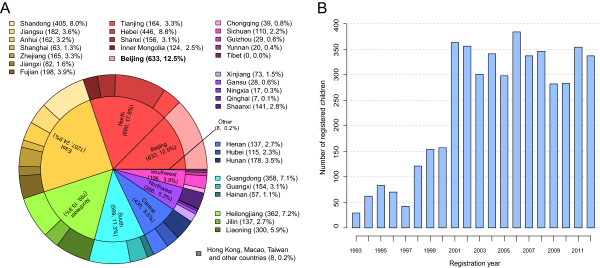
**Numbers and geographical distribution of** **the** **Beijing Stars and Rain Education Institute for Autism** **(SR) children,** **1993 to 2012. (A)** Geographical distribution of the SR children by region (inner) and province (outer). Beijing, as the capital of China, was listed separately from the northern region. **(B)** The number of children registered at the SR per year.

The 5,143 children registered at the SR had a male-to-female ratio of approximately 6.92:1. The male-to-female ratio was statistically significantly higher in Southern China (11.02:1) than in other regions (Fisher’s exact test, Benjamini and Hochberg false discovery rate (FDR) adjusted *P* value = 6.8e-3, [see Additional file 3: Table S5]). The 2,834 children with a parent-reported clinical diagnosis of ASD or ‘autistic tendencies’ had a male-to-female ratio of approximately 6.97:1. This ratio was higher than the estimates in many countries (approximately 4:1 to 5:1) but consistent with recent estimates in European and Chinese populations [45, 46]. This ratio was also higher in Southern China (8.82:1), but it was not statistically significant after FDR adjustment (Fisher’s exact test, Benjamini and Hochberg FDR adjusted *P* value =1.00, [see Additional file 3: Table S5]). The ratio did not show a statistically significant difference for different maternal or paternal education level (by Fisher’s exact test), and did not show a statistically significant trend in terms of diagnosis year or the age at diagnosis (by logistic regression) [see Additional file 3: Table S6, S7, S8].The SR children came from diverse geographical locations in China. A total of 70.9% were from economically developed regions such as the north, east and northeast, and 9.2% were from economically less developed regions such as the southwest and northwest (Figure 1A). Nearly half of the children were from the following six cities or provinces: Beijing (12.5%), Hebei (8.8%), Shandong (8.0%), Heilongjiang (7.2%), Guangdong (7.1%) and Liaoning (5.9%) (Figure 1A).As shown in Figure 1B, the number of children registered at the SR increased steadily prior to 2001, except for between 1996 and 1997. As mentioned in the Background section, in 1996, the SR changed its service mode. Since 1998, parents began to accept this new service mode, resulting in a steady increase in the numbers of registered children again (Figure 1B). In 2001, when the capacity of the SR was reached and other treatment centers in China began to operate, the number of children plateaued (Figure 1B).

### Characteristics of 100 autism intervention centers

**Figure 2:**
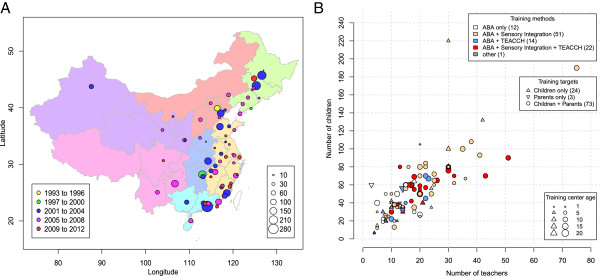
**Distribution and characteristics of 100 autism intervention centers. (A)** Cities in China are highlighted in weighted, colored circular dots, with dot size representing the total number of children being trained and colors indicating the year in which the first center was founded in the city. **(B)** Five properties of the intervention centers. As shown in the legend, the color of the symbol indicates the training methods used in the center, the shape represents the training targets and the size indicates the age of the center. Abbreviations: ABA, Applied Behavioral Analysis; TEACCH, Treatment and Education of Autistic and related Communication Handicapped Children.

Figure 2A and Additional file 4: Table S9 summarized the characteristics of the 100 autism intervention centers that participated in our survey. A total of 5,360 children were being trained in the 100 autism intervention centers at the time of the survey. Among them, 3,957 children had received a clinical diagnosis of ASD. Almost all of these centers provided services to children with ASD as well as to children with other developmental disabilities such as developmental delay, intellectual disabilities and language disorders. Ninety-seven of the centers opened after the year 2000. Seventy-six were located in the eastern, northeastern, northern and southern regions of China, which are economically more developed. Ninety-nine centers are private commercial entities or Non-Governmental Organizations; sixty-three of the centers were founded by parents of children with autism. These findings are consistent with the findings from previous studies of a smaller number of centers [9, 11, 12].

Among these 100 centers, 99 offered ABA intervention, 73 offered sensory integration intervention, and 36 offered the Treatment and Education of Autistic and related Communication Handicapped Children (TEACCH) program. Eighty-seven centers offered two or more intervention methods. Seventy-three centers trained both children and their parents, twenty-four centers focused only on child intervention, and three focused only on parent training (Figure 2B, [see Additional file 4: Table S9]). Sixty-seven centers trained both young children (under age 10) and older children (above age 10). Services for children over age 18 were available in only 12 centers. The number of staff at each center varied widely, ranging from 3 to 75, and positively correlated with the number of children, with a median staff-to-child ratio of 1:2.77 (Figure 2B, [see Additional file 4: Table S9]).

### Increasing number of hospitals making diagnoses of autism spectrum disorder

**Figure 3:**
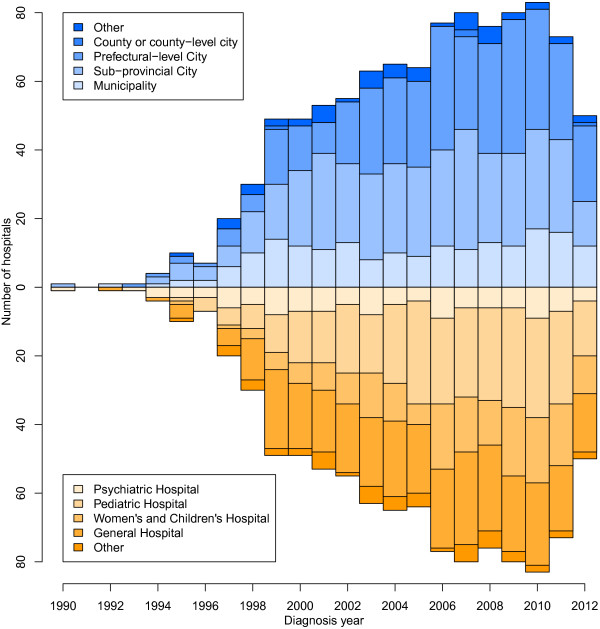
**From 1990 to 2012**, **the number of hospitals making diagnoses of autism spectrum disorder (ASD) increased.** The number of hospitals making diagnoses of ASD is shown for each year, grouped by the level of the cities in which they are located (upper panel) and the type of hospitals (lower panel).

The total number of hospitals where the SR children received their diagnoses of ASD or ‘autistic tendencies’ increased steadily over time, from just a few hospitals in the early 1990s to 267 hospitals at the time of study [see complete list in Additional file 5: Table S10]. In particular, the number of hospitals in prefecture-level cities and sub-provincial cities increased rapidly (Figure 3 Upper Panel, [see Additional file 6: Figure S2A]). Between 2007 and 2012, approximately half of the diagnoses were made by hospitals in municipalities, a quarter in sub-provincial cities and about a fifth in prefecture-level cities [see Additional file 7: Table S11]. Among these hospitals, 56.4% were located in the north, east, and northeast of China, with the most rapid increase taking place in the east. By contrast, the number of hospitals in the central and western regions, which are less developed, grew slowly [see Additional file 6: Figure S2C].

During the study period, a wider variety of hospitals began to make diagnoses. As shown in the lower panel of Figure 3 and Additional file 6: Figure S2B, in addition to psychiatric hospitals, a rapidly growing number of pediatric hospitals, women’s and children’s hospitals and general hospitals were making diagnoses. This increase might be driven by patient demand, as previous studies had found that most parents prefer to visit a pediatric or women’s and children’s hospital instead of a psychiatric hospital [11]. In recent years, the number of children diagnosed in pediatric hospitals was very close to the number diagnosed in psychiatric hospitals, accounting for approximately 35% of diagnoses [see Additional file 8: Table S12].

The five hospitals making the most diagnoses in our study sample were Peking University Sixth Hospital, Beijing Children’s Hospital, The Third Affiliated Hospital of Sun Yat-sen University, Nanjing Child Mental Health Research Center, and Tianjin Children’s Hospital [see Additional file 6: Figure S2D]. The number of children diagnosed at Peking University Sixth Hospital was the largest and continued to increase.

### Confirmation of parent-reported clinical diagnoses

Reassessment by ADI-R and re-diagnosis by clinical psychiatrists of 42 children confirmed that most of the parent-reported clinical diagnosis of ASD and ‘autistic tendencies’ were reliable, including 96.8% of children with a parent-reported clinical diagnosis of ASD and all children with ‘autistic tendencies’ (Table 1) [see ADI-R scores in Additional file 9: Table S13]. Therefore, further analyses were conducted using data from children with parent-reported clinical diagnosis of ASD or ‘autistic tendencies’ (n = 2,834).

**Table 1:** **Confirmation of parent**-**reported clinical diagnoses**

Group by reported diagnosis	Number of children in the group	Number (percent) of children re-diagnosed as ASD by ADI-R	Number (percent) of children re-diagnosed as ASD by child psychiatrists
ASD	31	30/31 (96.8%)	30/31 (96.8%)
Autistic tendencies	11	11/11 (100.0%)	11/11 (100.0%)
Total	42	41/42 (97.6%)	41/42 (97.6%)

Abbreviations: *ADI-R* Autism Diagnostic Interview, Revised, *ASD* autism spectrum disorder.

### Trends in the parent-reported clinical diagnosis of ‘autistic tendencies’

**Figure 4:**
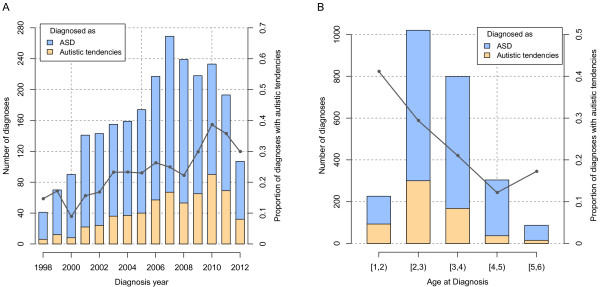
**Numbers and proportions of diagnoses of ‘** **autistic tendencies’,** **1998 to 2012.** The number of diagnoses of autism spectrum disorder (ASD) is shown in blue bars and the number of diagnoses of ‘autistic tendencies’ is shown in orange bars. The proportion of diagnoses of ‘autistic tendencies’ is represented by a gray line. The data were binned by year of diagnosis **(A)** and age at diagnosis **(B)**. Because the mean delay between the time of diagnosis and the time of application to the Beijing Stars and Rain Education Institute for Autism (SR) was approximately one year, most of the children who were diagnosed in 2012 had not yet applied; therefore, the number of diagnoses of ‘autistic tendencies’ declined in 2012. The children diagnosed before 1998 **(A)**, and children with age of less than 1 year at diagnosis **(B)** were excluded due to small sample sizes (less than 30).

Out of the 2,834 SR children, 2,222 (78.4%) had parent-reported clinical diagnosis of ASD and 612 (21.6%) of ‘autistic tendencies’. We found that the proportion of the diagnoses of ‘autistic tendencies’ had a statistically significant increase of 2.04-fold from 1998 to 2012, with an adjusted odds ratio of 2.36 per decade (95% CI: 1.78 to 3.12, *P* value of 2.3e-09, GEE model) (Figure 4A, [see Additional file 1: Table S1]). Additionally, the proportion was statistically significantly higher in younger children, with 31.5% for those diagnosed between 1 and 3 years old versus 18.5% for those diagnosed between 3 and 6 years old, with an adjusted odds ratio of 1.60 for every year of decrease in the age at diagnosis (95% CI: 1.42 to 1.81, *P* value = 5.0e-14, GEE model) (Figure 4B, [see Additional file 1: Table S1]).

### The mean age at first diagnosis of autism spectrum disorder and ‘autistic tendencies’ declined over the past two decades

**Figure 5:**
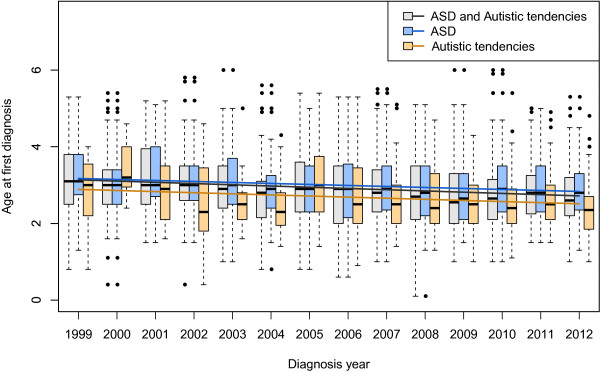
**Decline in age at first diagnosis**, **1999 to 2012.** The distributions of age at first diagnosis are presented for each year. The thick band inside the box is the median, and the bottom and top of the box are the first quantile (Q1) and the third quantile (Q3). The ends of the whiskers represents data within 1.5 *interquartile range (IQR) from the lower quantile (Q1) or the upper quantile (Q3), where IQR is equal to Q3 minus Q1. The fitted simple linear regression lines are highlighted in different colors (black line: slope = -3.2e-02 (95% confidence interval (CI): -4.3e-02 to -2.2e-02), *P* value = 3.2e-09, blue line: slope = -2.6e-02 (95% CI: -3.8e-02 to -1.4e-02), *P* value = 3.2e-05, orange line: slope = -2.9e-02 (95% CI: -5e-02 to -7.8e-03), *P* value = 7.3e-03). Abbreviation: ASD, autism spectrum disorder.

We observed statistically significant decline over time in the mean age at first diagnosis for the SR children with a parent-reported clinical diagnosis of ASD, ‘autistic tendencies’, and ASD-or-autistic tendencies, respectively (Figure 5). After controlling for ‘autistic tendencies’, the multiple linear regression model showed that the mean age at first diagnosis had a statistically significant decrease of 0.27 years per decade (95% CI: 0.16 to 0.37) (*P* value = 7.8e-07, [see Additional file 1: Table S2]).

### Higher parental education levels were associated with lower age at first diagnosis

**Figure 6:**
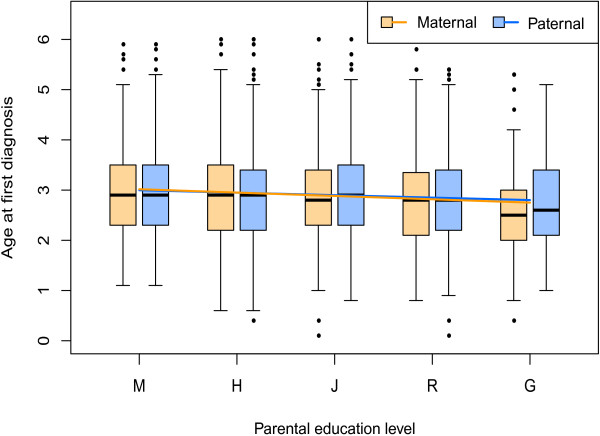
**The correlation between age at first diagnosis and parental education level.** Box plots show the age at first diagnosis grouped by maternal education level and paternal education level. The thick band inside the box is the median, and the bottom and top of the box are the first quantile (Q1) and the third quantile (Q3). The ends of the whiskers represents data within 1.5 *interquartile range (IQR) from the lower quantile (Q1) or the upper quantile (Q3), where IQR is equal to Q3 minus Q1. The fitted simple linear regression lines are highlighted in different colors (maternal orange line: slope = -6.7e-02 (95% confidence interval (CI): -1e-01 to -3.3e-02), *P* value = 1.3e-04, paternal blue line: slope = -4.9e-02 (95% CI: -8.2e-02 to -1.6e-02), *P* value = 3.9e-03). Abbreviations: M, middle school or lower; H, high school; J, junior college; R, regular college; G, graduate.

Previous studies in other countries had found that higher parental education levels were associated with earlier ages at first diagnosis of ASD in the children [47–49]. Our analysis of the SR data revealed a similar trend (Figure 6). The multiple linear regression models showed that both maternal and paternal education level had a statistically significant negative correlation with the child’s age at first diagnosis after adjusting for diagnosis year and the age at childbirth (*P* value = 1.3e-03 and 3.9e-02, respectively) [see Additional file 1: Table S3]. The correlation of maternal education level with early diagnosis appeared to be more statistically significant than paternal education level; however, because the education levels of the two parents are strongly correlated (Spearman’s rank correlation coefficient rho = 0.75, *P* value <2.2e-16), their separate influence on the age at first diagnosis was difficult to measure.

### Increase in parental age at childbirth

**Figure 7:**
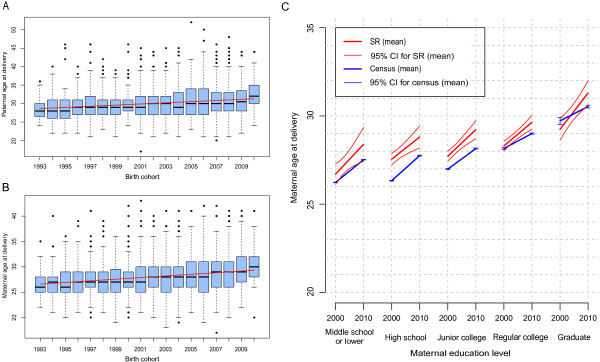
**Trend in parental age at childbirth and comparison with the general population. (A, B)** Box plots for paternal **(A)** and maternal **(B)** ages at childbirth between 1993 and 2010. The thick band inside the box is the median, and the bottom and top of the box are the first quantile (Q1) and the third quantile (Q3). The ends of the whiskers represents data within 1.5 *interquartile range (IQR) from the lower quantile (Q1) or the upper quantile (Q3), where IQR is equal to Q3 minus Q1. The fitted simple linear regression lines for parental age at childbirth are highlighted in red (paternal line: slope =1.6e-01 (95% confidence interval (CI): 1.2e-01 to 1.9e-01), *P* value =1.5e-15, maternal line: slope = 1.6e-01 (95% CI: 1.3e-01 to 1.9e-01), *P* value <2e-16). **(C)** Comparison of maternal age at childbirth of mothers of the Beijing Stars and Rain Education Institute for Autism (SR) children with a reported diagnosis of autism spectrum disorder (ASD) or ‘autistic tendencies’ and mothers in the general population. Fitted simple linear regression lines based on the SR data for maternal age at childbirth between 2000 and 2010 are represented by red thick solid lines and grouped by maternal education level, with 95% CI for the mean shown as red thin solid curves. The blue thick solid lines, based on 2000 and 2010 Chinese census data, connect the mean maternal ages at childbirth and are grouped by maternal education level. The blue thin solid segments show the 95% CI for the mean of each subset calculated using the mean and standard error.

As shown in Figure 7A and B, the mean paternal and maternal ages at childbirth of the SR children with a parent-reported clinical diagnosis of ASD or ‘autistic tendencies’ increased from 28.53 in 1993 to 32.52 in 2010 and from 26.62 in 1993 to 30.15 in 2010, respectively. The proportions of fathers and mothers aged 35 years or older at childbirth increased from 7.5% in 1993 to 29.6% in 2010 and from 2.6% in 1993 to 11.1% in 2010, respectively. The multiple linear regression showed that the parental age at childbirth, after adjusting for education level, increased statistically significantly, with a *P* value of 4.0e-14 for paternal age and a *P* value <2e-16 for maternal age. The paternal and maternal age at childbirth increased by 1.47 (95% CI: 1.09 to 1.84) and 1.48 (95% CI: 1.15 to 1.81) years per decade, respectively [see Additional file 1: Table S4].

Using data from the 2000 and 2010 Chinese censuses, we observed a similar increase in the mean maternal age at childbirth in the general population from 26.29 in 2000 to 27.68 in 2010 (Table 2). A total of 3.8% and 13.7% of births in 2000 and 2010, respectively, were to mothers age 35 years or older. A similar increase was observed in other developed countries according to their national statistics reports, many with even higher reproductive ages than in China [50, 51]. In comparison, when we restricted our study population of the SR children to the time period coinciding with the 2000 and 2010 Chinese national censuses, the mean age of the mothers who gave birth to the SR children diagnosed with ASD or ‘autistic tendencies’ was statistically significantly higher than that of mothers in the general population - on average 1.40 years higher in 2000 and 2.10 years higher in 2010 (Table 2). Even after grouping the samples by maternal education level, we still observed an elevated maternal age of the SR study population compared to the general population for all education levels except for the highest level, at which the ages were similar (Figure 7C).

**Table 2:** **Comparison of mean maternal age at birth of the Beijing Stars and Rain Education Institute for Autism** (**SR) children with a parent**-**reported diagnosis of autism spectrum disorder (ASD) or** ‘**autistic tendencies**’ **and that of general population**

Year	SR children		General population		***P***value derived from Mann-Whitney test
	n	Mean (standard deviation) age at childbirth	n	Mean (standard deviation) age at childbirth	
2000	134	27.69 (3.33)^a^	1181803	26.29 (4.12)^c^	1.63e-07
2010	64	29.78 (3.71)^b^	1157270	27.68 (5.98)^d^	5.93e-06

^a^Maternal age at birth of 134 children of the SR with a parent-reported diagnosis of ASD or ‘autistic tendencies’ born between 1 November 1999 and 31 October 2000.

^b^Maternal age at birth of 64 children of the SR with a parent-reported diagnosis of ASD or ‘autistic tendencies’ born between 1 November 2009 and 31 October 2010.

^c^Maternal age at birth of 1,181,803 children between 1 November 1999 and 31 October 2000 from China’s 2000 census data.

^d^Maternal age at birth of 1,157,270 children between 1 November 2009 and 31 October 2010 from China’s 2010 census data.

## Discussion

We report here the first statistical analysis of twenty years of data from China’s first autism intervention center and survey results from 100 autism intervention centers. It was estimated that there are about 1,000 autism intervention centers in China at present (H Wen, personal communications). The SR and the total of 100 autism intervention centers we studied represent a subset, but not all, of the intervention centers in China. Because no unbiased, nationwide surveys in China of children with ASD or autism intervention centers have been reported yet, we cannot quantify the exact biases in our studied population, but there were several potential biases and limitations. First, because the SR only accepts children age six and younger, children with higher function were under-represented, as these children have been found by previous studies to be diagnosed at higher ages [52, 53]. Second, children who received training were only a subset of all children diagnosed, and children who were diagnosed were only a subset of all children who were affected by ASD. Lastly, state-owned autism intervention centers were under-represented in the 100 centers we studied. Larger and unbiased nationwide surveys are necessary for replicating our findings. Nevertheless, compared to previous publications on ASD diagnosis and intervention in China, our study covered a much larger number of patients and centers and performed more rigorous statistical tests.

Since the Diagnostic and Statistical Manual of Mental Disorders, Fifth edition (DSM-5) was released in May 2013 and our studied population was registered at the SR before 2012, diagnoses were made according to the CCMD-3, which was based on the DSM-IV and ICD-10. Re-diagnoses of the 42 children were made according to the DSM-IV-TR. In our studied population, six children had a diagnosis of Rett syndrome, which would not have been diagnosed as ASD by the DSM-5. However for consistency we still included them in our analyses. This small number of children should not have affected our results. The 42 children who we re-diagnosed were not randomly selected from our total sample, but instead they were the subset of children who also participated in our Autism Genetic Research Project, which began in early 2012. All of these children registered at the SR in 2011 and 2012. Thus, they did not represent the total sample from the SR, especially the children registered before 2011. A larger, randomized evaluation of clinical diagnosis in China needs to be conducted.

Among the 2,222 children at the SR with a parent-reported diagnosis of ASD, 34 children had a reported diagnosis of high functioning autism, 20 children had a reported diagnosis of Asperger Syndrome, and no children were reported with PDD-NOS. For the 31 children in the ‘ASD’ group that we re-diagnosed, 30 were found to have ASD, including classic autistic disorders (n = 22), high functioning autism (n = 2), Asperger Syndrome (n = 5) and Rett syndrome (n = 1) [see Additional file 9: Table S13]. In contrast, among these 31 children, only two children had a parent-reported clinical diagnosis of high functioning autism, and all others were reported as having classic autistic disorders. This indicated that there was insufficient distinction between classic autism, Asperger Syndrome, and PDD-NOS in the current clinical practice in China, which was consistent with the previous report [9].

In the statistical analysis of the SR data, to be stringent, we focused only on 2,834 children with a parent-reported clinical diagnosis of ASD or ‘autistic tendencies’. The ‘other’ children included 203 with a parent-reported clinical diagnosis of other neuropsychiatric disorders and 2,106 missing parent-reported clinical diagnosis. Among the 2,106 children missing a parent-reported clinical diagnosis, 18 participated in our other project, the Autism Genetic Research Project. We found that 13 (approximately 70%) of children in fact had a clinical diagnosis of ASD (11 children) or ‘autistic tendencies’ (2 children) that the parent failed to report. Another four (22.2%) children without a parent-reported diagnosis did not receive an official clinical diagnosis but had behavior impairments and were suggested by doctors or teachers to seek early intervention without waiting for official diagnoses to be made. They were confirmed by our re-diagnosis to indeed have ASD. Thus in total, approximately 90% of the SR children with missing parent-reported diagnosis may in fact have ASD. The SR staff routinely reassessed all children at the beginning of the intervention and found that over 90% of all children who enrolled at the SR satisfied the clinical criteria for ASD, which was consistent with findings from our re-diagnoses of the small subset. This pattern was also consistent with a previous study reported that 26 of 100 children with ASD in China obtained their autism ‘diagnosis’ in autism intervention centers instead of hospitals [11].

The number and proportion of diagnoses of ‘autistic tendencies’ increased statistically significantly, especially for children diagnosed at a younger age. All 11 children in the ‘autistic tendencies’ group that we re-diagnosed were found to have ASD, including classic autistic disorders (n = 9), high functioning autism (n = 1), and Asperger Syndrome (n = 1) [see Additional file 9: Table S13]. Our sample size was small, but it did reflect the tendency of clinicians to make ‘autistic tendencies’ diagnosis in China that was also reported by previous studies [9]. Making an accurate diagnosis of ASD in children under age three, especially high-functioning children, may be challenging. Doctors in China tend to make a diagnosis of ‘autistic tendencies’ and to encourage parents to seek early intervention instead of waiting for a definitive diagnosis [9]. Furthermore, because there is not enough special support for patients diagnosed with ASD and regular public schools often reject children with ASD diagnosis (even children with high-functioning autism), doctors in China are sometimes reluctant to label a child as having ASD if he or she is young or exhibits less severe symptoms and are inclined to give the diagnoses of ‘autistic tendencies’ instead [9].

The hospitals where the SR children received their ASD or ‘autistic tendencies’ diagnosis increased in both number and diversity over the two decades. However, despite the progress, approximately half of the children obtained their diagnoses in psychiatric and pediatric hospitals in large cities. Few hospitals in small cities such as counties or county-level cities were able to diagnose ASD. The ASD diagnostic capabilities of doctors in small cities still need to be improved. In addition, even though the number of autism intervention centers had increased significantly since 2000, the approximately 1000 autism intervention centers at present were still far from enough considering the large population of children affected with ASD in China. More training services, especially in under-developed regions, are needed to help this large population.

## Conclusions

Our statistical analyses revealed trends in ASD diagnoses in 20 years of data from the SR and from the current situation of ASD intervention at 100 autism centers. We highlighted encouraging trends such as an increase in the number of hospitals making ASD diagnoses, an increase in the number of ASD intervention centers, and a decrease in the average age at first diagnosis of ASD. However, challenges still remain, such as a limited number of hospitals and intervention centers in under-developed regions.

## Electronic supplementary material

Additional file 1: Table S1-S4: The model formula, sample size and fitted coefficients for multiple regression models or GEE. The model formula was written in R-like style, which put the response variable left of ' ~ ' and the predictor variables right of ' ~ ', concatenated by ' + '. VIF, variance inflation factor. (XLS 44 KB)

Additional file 2: Figure S1: Residual plots for multiple linear regression models reported in Table S2-S4. (Left: residuals plotting against the fitted value; right: normal QQ plot of residuals). (PDF 2 MB)

Additional file 3: Table S5-S8: The male and female count and ratio of children registered at the Beijing Stars and Rain Education Institute for Autism (SR). There were 5,143 children in total, among which 2,834 children had a parent-reported clinical diagnosis of autism spectrum disorder (ASD) or ‘autistic tendencies’. For analyses related to diagnosis, after applying the criteria on the age at registration, there remained 2,138 children. (XLS 37 KB)

Additional file 4: Table S9: The list of 100 autism intervention centers that participated in our survey. (XLS 61 KB)

Additional file 5: Table S10: The list of hospitals making diagnoses of autism spectrum disorder (ASD) obtained from the Beijing Stars and Rain Education Institute for Autism (SR) data. (XLS 56 KB)

Additional file 6: Figure S2: Trends of different groups of hospitals making diagnoses of autism spectrum disorder (ASD). (A) Line chart for the number of hospitals during 1990-2012 grouped by the level of cities in which they are located, and (B) the type of hospital. (C) The number of hospitals located in different regions during 1990-2012. (D) The number of diagnoses made in the top five hospitals during 1990-2012. Because the mean delay between the time of diagnosis and the time of application to the Beijing Stars and Rain Education Institute for Autism (SR) was approximately one year, most of the children who were diagnosed in 2012 have not yet applied; therefore, the number of hospitals declined in 2012. (PDF 1 MB)

Additional file 7: Table S11: The numbers of diagnoses grouped by the level of cities in which the hospitals are located and diagnosis year. (XLS 39 KB)

Additional file 8: Table S12: The numbers of diagnoses grouped by the hospital type and diagnosis year. (XLS 39 KB)

Additional file 9: Table S13: The parent-reported diagnosis, Autism Diagnostic Interview, Revised (ADI-R) score and rediagnosis results given by child psychiatrists for 42 children that we reassessed. (XLS 25 KB)

## Abbreviations

ABA: Applied Behavioral Analysis
ADI-R: Autism Diagnostic Interview, Revised
ASD: autism spectrum disorder
CCMD-3: Chinese Classification and Diagnosis Criteria of Mental Disorders, 3^rd^ Edition
CI: confidence interval
DSM-IV: Diagnostic and Statistical Manual of Mental Disorders, Fourth edition
DSM-IV-TR: Diagnostic and Statistical Manual of Mental Disorders, Fourth edition, text revised
DSM-5: Diagnostic and Statistical Manual of Mental Disorders, Fifth edition
FDR: false discovery rate
GEE: Generalized Estimating Equations
ICD-10: International Statistical Classification of Diseases and Related Health Problems, 10th Revision
IQR: interquartile range
PDD-NOS: Pervasive Developmental Disorder, Not Otherwise Specified
SR: Beijing Stars and Rain Education Institute for Autism
TEACCH: treatment and education of autistic and related communication handicapped children
VIF: variance inflation factor.

## References

[1] American Psychiatric Association (2013). Diagnostic and Statistical Manual of Mental Disorders.

[2] Tao K (1982). The problems of diagnosis and classification of infantile autism. J Chinese Neuropsychiatry.

[3] Leading Group of the Second China National Sample Survey on Disability & National Bureau of Statistics of the People’s Republic of China (2006). Communique on major statistics of the second China national sample survey on disability. Chin J Rehabil Theory Pract.

[4] CDC (2009). Prevalence of autism spectrum disorders - Autism and Developmental Disabilities Monitoring Network, United States, 2006. MMWR Surveill Summ.

[5] CDC (2012). Prevalence of autism spectrum disorders--Autism and Developmental Disabilities Monitoring Network, 14 sites, United States, 2008. MMWR Surveill Summ.

[6] CDC (2014). Prevalence of autism spectrum disorder among children aged 8 years - autism and developmental disabilities monitoring network, 11 sites, United States, 2010. MMWR Surveill Summ.

[7] Blumberg SJ, Bramlett MD, Kogan MD, Schieve LA, Jones JR, Lu MC (2013). Changes in prevalence of parent-reported autism spectrum disorder in school-aged U.S. children: 2007 to 2011-2012. Natl Health Stat Report.

[8] Kim YS, Leventhal BL, Koh YJ, Fombonne E, Laska E, Lim EC, Cheon KA, Kim SJ, Kim YK, Lee H, Song DH, Grinker RR (2011). Prevalence of autism spectrum disorders in a total population sample. Am J Psychiatry.

[9] Huang AX, Jia M, Wheeler JJ (2013). Children with Autism in the People’s Republic of China: Diagnosis, Legal Issues, and Educational Services. J Autism Dev Disord.

[10] H M (2003). The beginnings of inclusion in the People’s Republic of China. Res Pract Pers Severe Disabil.

[11] Sun X, Allison C, Auyeung B, Baron-Cohen S, Brayne C (2013). A review of healthcare service and education provision of Autism Spectrum Condition in mainland China. Res Dev Disabil.

[12] McCabe H (2013). Bamboo shoots after the rain: Development and challenges of autism intervention in China. Autism.

[13] Sun X, Allison C, Auyeung B, Matthews FE, Murray S, Baron-Cohen S, Brayne C (2013). Service provision for autism in mainland China: a service providers’ perspective. Res Dev Disabil.

[14] Chinese Psychiatry Association (2001). The Chinese Classification and Diagnosis Criteria of Mental Disorder (3rd ed., CCMD-3rd).

[15] American Psychiatric Association (1994). The Diagnostic and Statistical Manual of Mental Disorders (4th ed., DSM-4th).

[16] World Health Organization (1993). The International Classification of Diseases (10th ed., ICD-10th).

[17] McCabe H, Tian H (2001). Early intervention for children with autism in the people’s republic of china: a focus on parent training. J Int Spec Needs Educ.

[18] **The Development of Beijing Stars and Rain**. [http://www.guduzh.org.cn/tabid/189/language/zh-CN/Default.aspx]

[19] Lovaas OI (1987). Behavioral treatment and normal educational and intellectual functioning in young autistic children. J Consult Clin Psychol.

[20] Harris SL, Handleman JS (2000). Age and IQ at intake as predictors of placement for young children with autism: a four- to six-year follow-up. J Autism Dev Disord.

[21] Fenske EC, Zalenski S, Krantz PJ, McClannahan LE (1985). Age at intervention and treatment outcome for autistic children in a comprehensive intervention program. Anal Interv Dev Disabil.

[22] Lai MC, Lombardo MV, Baron-Cohen S (2014). Autism. Lancet.

[23] Koegel LK, Koegel RL, Ashbaugh K, Bradshaw J (2014). The importance of early identification and intervention for children with or at risk for autism spectrum disorders. Int J Speech Lang Pathol.

[24] Durkin MS, Maenner MJ, Newschaffer CJ, Lee LC, Cunniff CM, Daniels JL, Kirby RS, Leavitt L, Miller L, Zahorodny W, Schieve LA (2008). Advanced parental age and the risk of autism spectrum disorder. Am J Epidemiol.

[25] Reichenberg A, Gross R, Weiser M, Bresnahan M, Silverman J, Harlap S, Rabinowitz J, Shulman C, Malaspina D, Lubin G, Knobler HY, Davidson M, Susser E (2006). Advancing paternal age and autism. Arch Gen Psychiatry.

[26] Croen LA, Najjar DV, Fireman B, Grether JK (2007). Maternal and paternal age and risk of autism spectrum disorders. Arch Pediatr Adolesc Med.

[27] King MD, Fountain C, Dakhlallah D, Bearman PS (2009). Estimated autism risk and older reproductive age. Am J Public Health.

[28] Grether JK, Anderson MC, Croen LA, Smith D, Windham GC (2009). Risk of autism and increasing maternal and paternal age in a large north American population. Am J Epidemiol.

[29] Shelton JF, Tancredi DJ, Hertz-Picciotto I (2010). Independent and dependent contributions of advanced maternal and paternal ages to autism risk. Autism Res.

[30] Hultman CM, Sparen P, Cnattingius S (2002). Perinatal risk factors for infantile autism. Epidemiology.

[31] Larsson HJ, Eaton WW, Madsen KM, Vestergaard M, Olesen AV, Agerbo E, Schendel D, Thorsen P, Mortensen PB (2005). Risk factors for autism: perinatal factors, parental psychiatric history, and socioeconomic status. Am J Epidemiol.

[32] McGrath JJ, Petersen L, Agerbo E, Mors O, Mortensen PB, Pedersen CB (2014). A comprehensive assessment of parental age and psychiatric disorders. JAMA psychiatry.

[33] Idring S, Magnusson C, Lundberg M, Ek M, Rai D, Svensson AC, Dalman C, Karlsson H, Lee BK (2014). Parental age and the risk of autism spectrum disorders: findings from a Swedish population-based cohort. Int J Epidemiol.

[34] Zhang X, Lv CC, Tian J, Miao RJ, Xi W, Hertz-Picciotto I, Qi L (2010). Prenatal and perinatal risk factors for autism in China. J Autism Dev Disord.

[35] **The Heart Alliance**. [http://www.guduzh.org.cn/tabid/264/Default.aspx]

[36] Clancy H, Dugdale A, Rendle-Short J (1969). The diagnosis of infantile autism. Dev Med Child Neurol.

[37] Lord C, Rutter M, Le Couteur A (1994). Autism Diagnostic Interview-Revised: a revised version of a diagnostic interview for caregivers of individuals with possible pervasive developmental disorders. J Autism Dev Disord.

[38] American Psychiatric Association (2000). Diagnostic and Statistical Manual of Mental Disorders (4th ed., text rev.).

[39] Fischbach GD, Lord C (2010). The Simons Simplex Collection: a resource for identification of autism genetic risk factors. Neuron.

[40] Risi S, Lord C, Gotham K, Corsello C, Chrysler C, Szatmari P, Cook EH, Leventhal BL, Pickles A (2006). Combining information from multiple sources in the diagnosis of autism spectrum disorders. J Am Acad Child Adolesc Psychiatry.

[41] Zuur A, Ieno EN, Walker N, Saveliev AA, Smith GM (2009). Mixed Effects Models and Extensions in Ecology with R.

[42] Halekoh U, Hojsgaard S, Yan J (2006). The R package geepack for generalized estimating equations. J Stat Softw.

[43] **The Fifth National Census Data (2000)**. [http://www.stats.gov.cn/tjsj/pcsj/rkpc/5rp/index.htm]

[44] **The Sixth National Census Data (2010)**. [http://www.stats.gov.cn/tjsj/pcsj/rkpc/6rp/indexch.htm]

[45] Whiteley P, Todd L, Carr K, Shattock P (2010). Gender Ratios in Autism, Asperger Syndrome and Autism Spectrum Disorder. Autism Insights.

[46] Wong VC, Hui SL (2008). Epidemiological study of autism spectrum disorder in China. J Child Neurol.

[47] Fountain C, King MD, Bearman PS (2011). Age of diagnosis for autism: individual and community factors across 10 birth cohorts. J Epidemiol Community Health.

[48] Goin-Kochel RP, Mackintosh VH, Myers BJ (2006). How many doctors does it take to make an autism spectrum diagnosis?. Autism.

[49] Rosenberg RE, Landa R, Law JK, Stuart EA, Law PA (2011). Factors affecting age at initial autism spectrum disorder diagnosis in a national survey. Autism Res Treat.

[50] Martin J, Hamilton B, Sutton P (2006). Births: final data for 2004. Natl Vital Stat Rep.

[51] Mathews T, Hamilton B (2002). Mean age of mother, 1970-2000. Natl Vital Stat Rep.

[52] Mandell DS, Novak MM, Zubritsky CD (2005). Factors associated with age of diagnosis among children with autism spectrum disorders. Pediatrics.

[53] Howlin P, Asgharian A (1999). The diagnosis of autism and Asperger syndrome: findings from a survey of 770 families. Dev Med Child Neurol.

