# Supplementary material for: Statistical analysis of twenty years (1993 to 2012) of data from mainland China’s first intervention center for children with autism spectrum disorder
Source: Mol Autism. 2014 Nov 12;5:52. doi: 10.1186/2040-2392-5-52 (PMC4332440; doi:10.1186/2040-2392-5-52)

A

age_at_first_diagnosis
~ first_diagnosis_year + first_diagnosis_is_autistic_tendencies

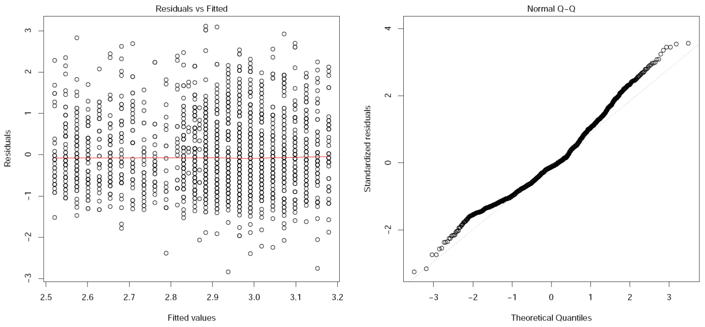

B

age_at_first_diagnosis ~ first_diagnosis_year
+ maternal(paternal)_age_at_childbirth + maternal(paternal)_education_level

(maternal) (paternal)

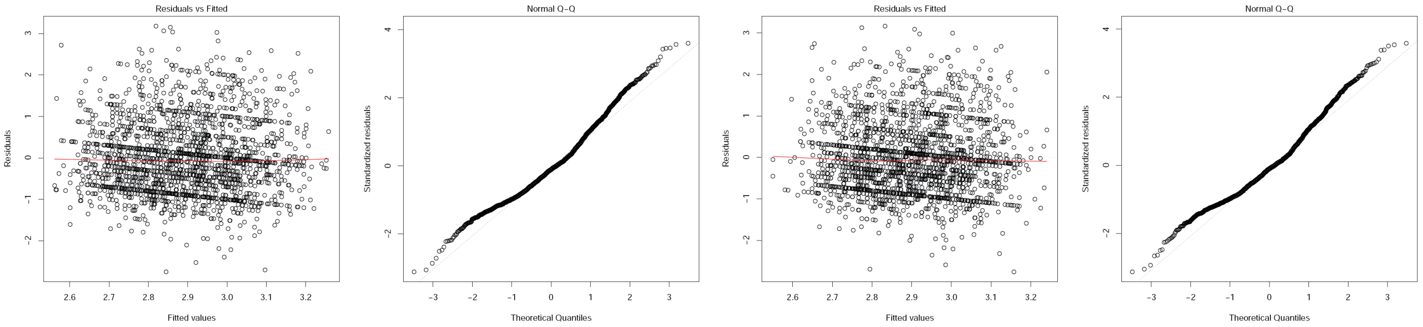

C

maternal(paternal)_age_at_childbirth
~ child_birth_year + maternal(paternal)_education_level

(maternal) (paternal)

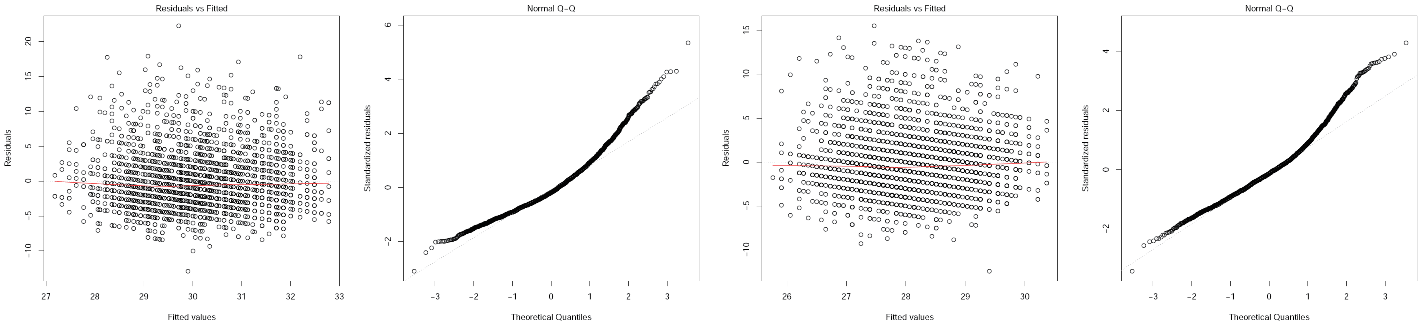

Supplement: Supplementary file 2 — Additional file 2: Figure S1: Residual plots for multiple linear regression models reported in Table S2-S4. (Left: residuals plotting against the fitted value; right: normal QQ plot of residuals). (PDF 2 MB) [file 13229_2014_154_MOESM2_ESM.pdf]
