# Supplementary figures and images for: Statistical analysis of twenty years (1993 to 2012) of data from mainland China’s first intervention center for children with autism spectrum disorder
Source: Mol Autism. 2014 Nov 12;5:52. doi: 10.1186/2040-2392-5-52 (PMC4332440; doi:10.1186/2040-2392-5-52)

A

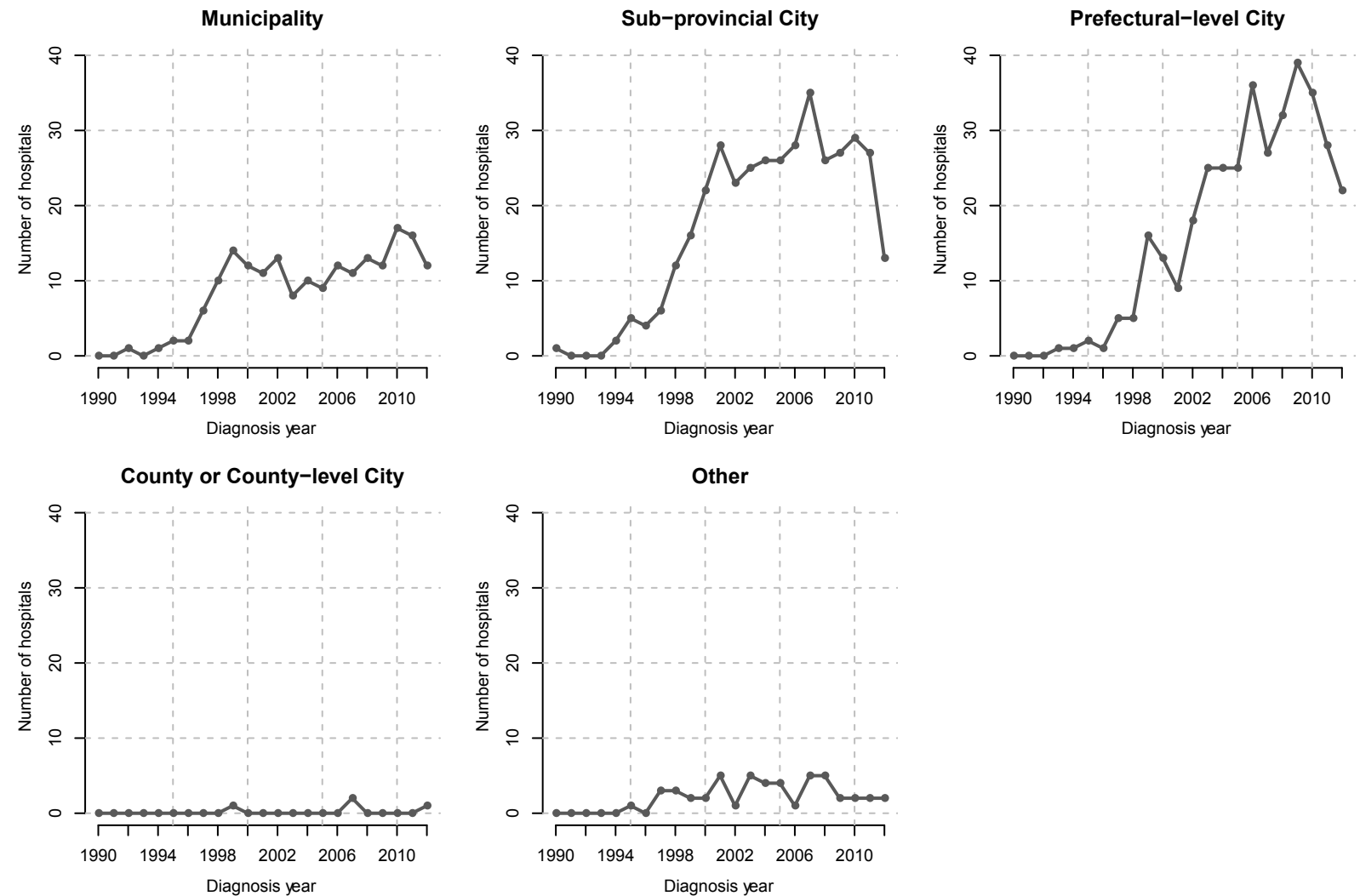

B

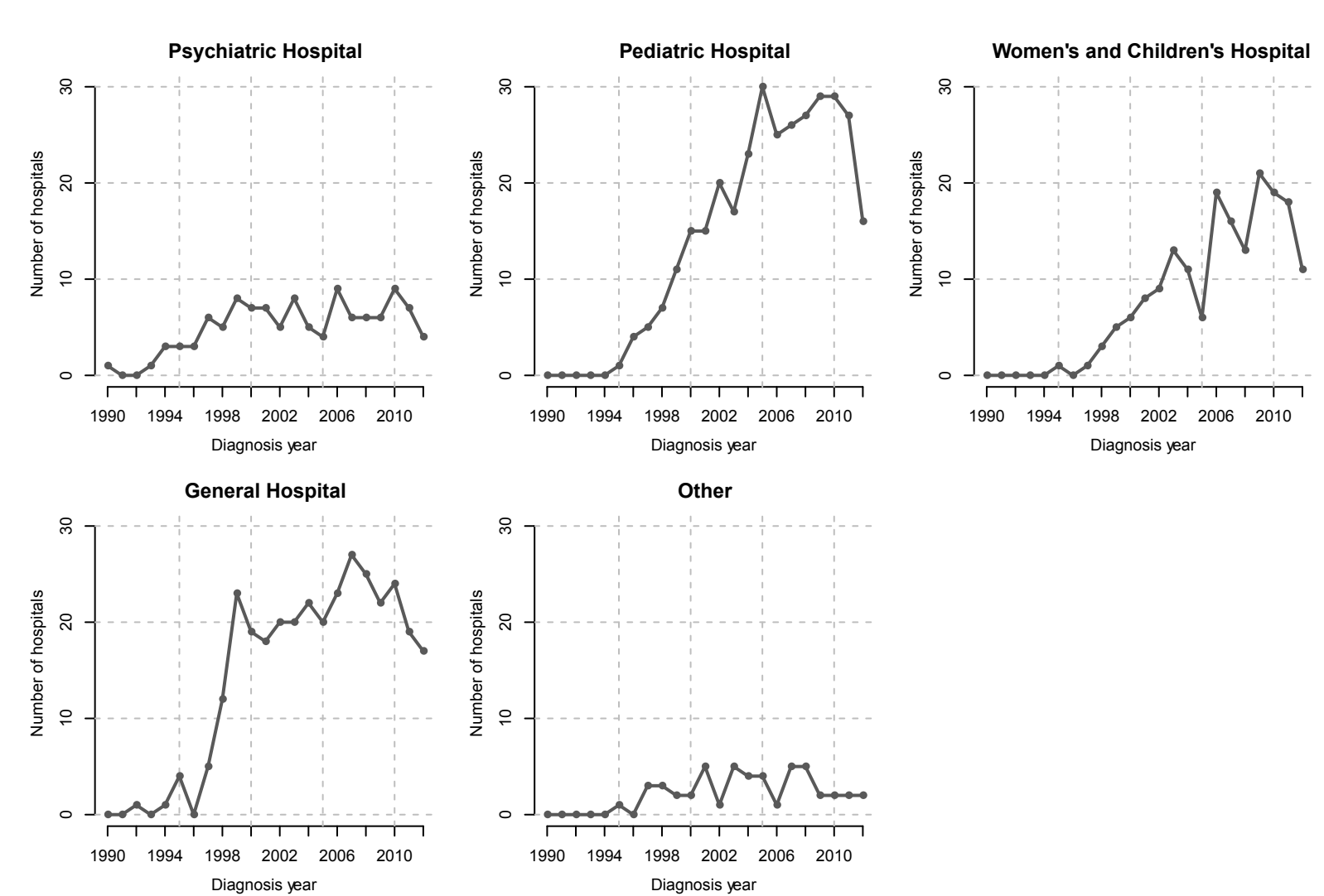

C

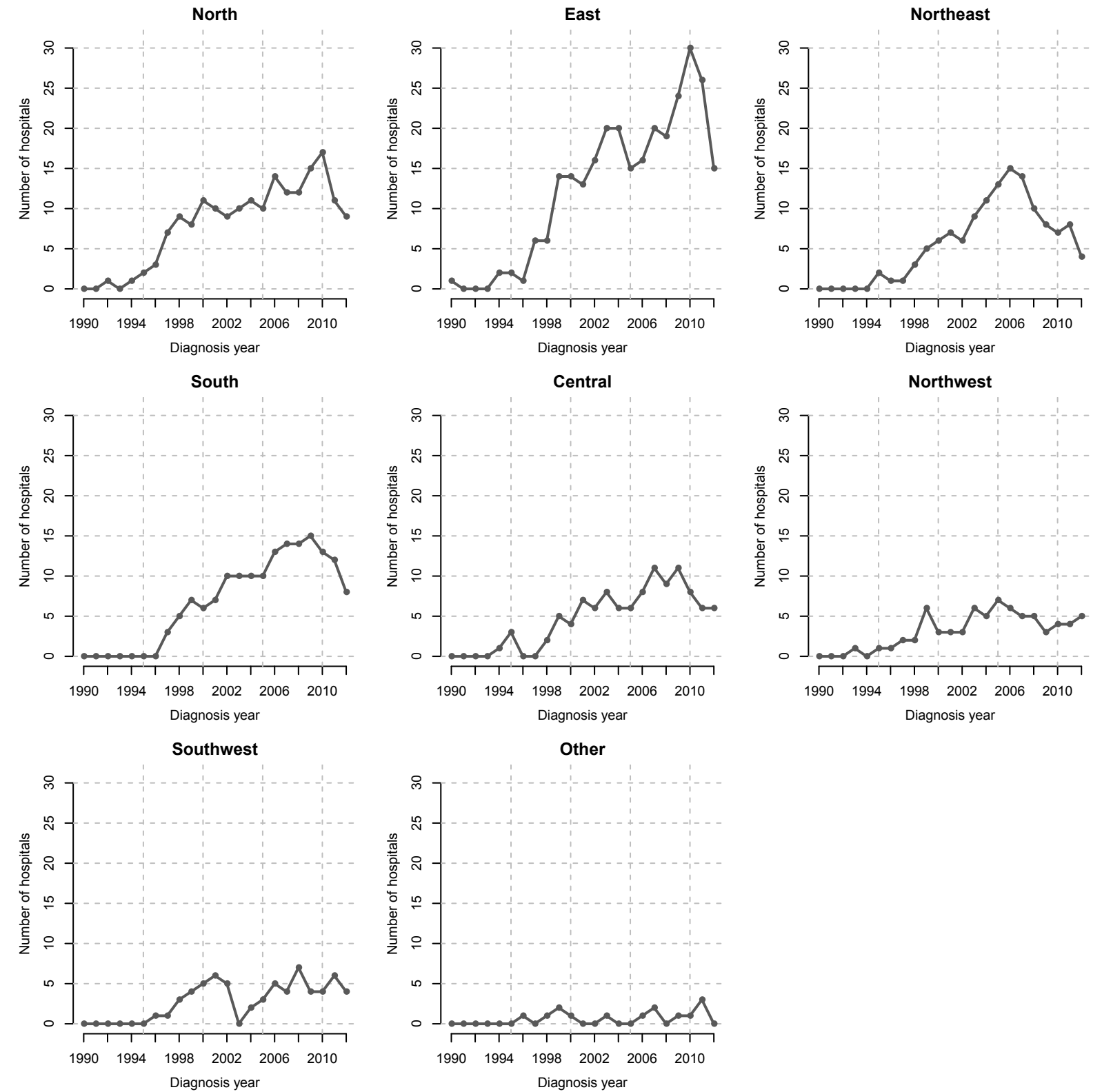

D

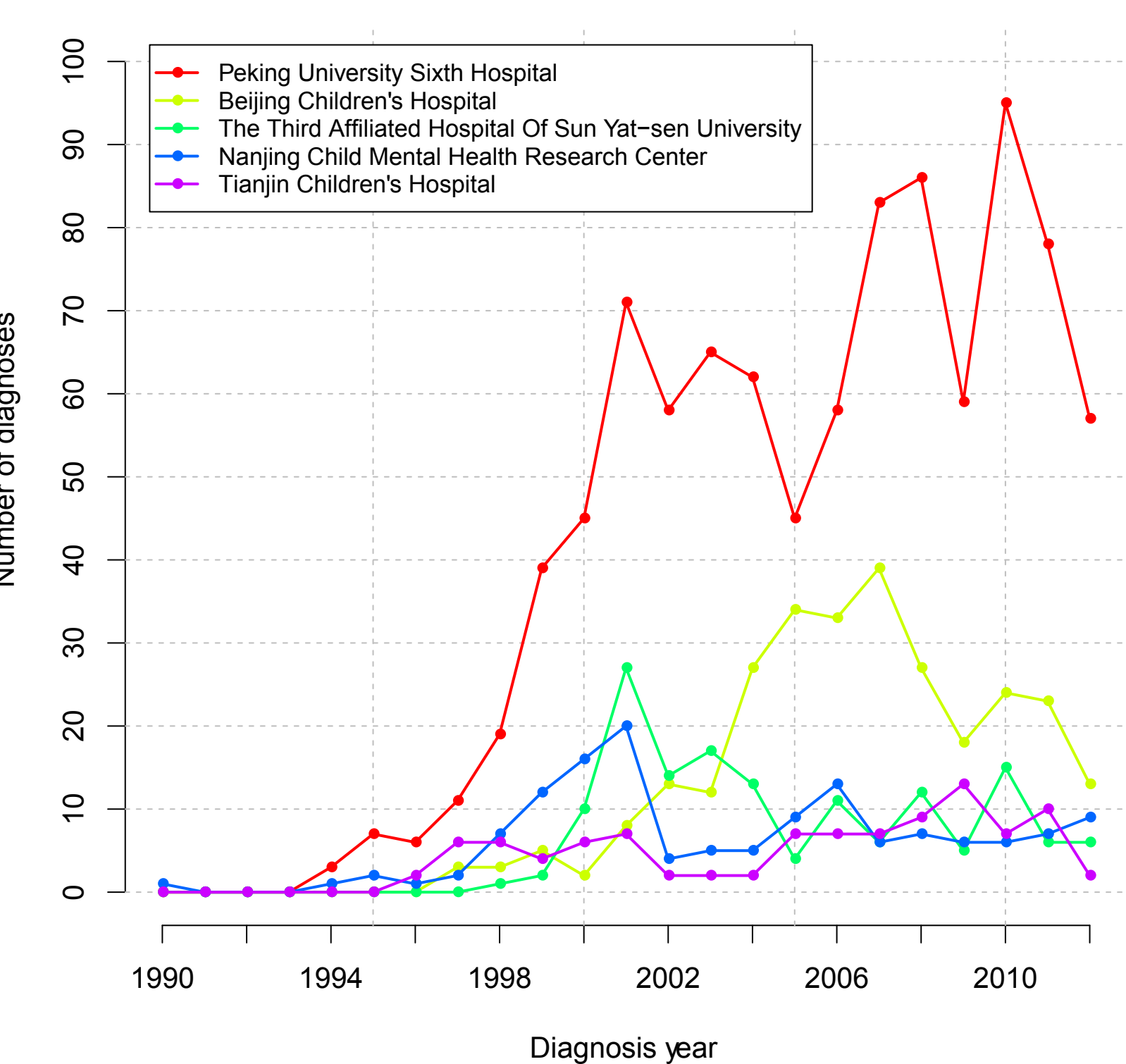

Supplement: Supplementary file 6 — Additional file 6: Figure S2: Trends of different groups of hospitals making diagnoses of autism spectrum disorder (ASD). (A) Line chart for the number of hospitals during 1990-2012 grouped by the level of cities in which they are located, and (B) the type of hospital. (C) The number of hospitals located in different regions during 1990-2012. (D) The number of diagnoses made in the top five hospitals during 1990-2012. Because the mean delay between the time of diagnosis and the time of application to the Beijing Stars and Rain Education Institute for Autism (SR) was approximately one year, most of the children who were diagnosed in 2012 have not yet applied; therefore, the number of hospitals declined in 2012. (PDF 1 MB) [file 13229_2014_154_MOESM6_ESM.pdf]
